# Supplementary material for: Toxoplasma TgATG9 is critical for autophagy and long-term persistence in tissue cysts
Source: eLife. 2021 Apr 27;10:e59384. doi: 10.7554/eLife.59384 (PMC8128441; doi:10.7554/eLife.59384)
Supplement: Supplementary file 1. [file elife-59384-supp1.docx]

Supplementary File 1. Primers used in this study.

| **Primer Name** | **Forward Primer  (5’ – 3’)** | **Reverse Primer**  **(5’ – 3’)** |
| --- | --- | --- |
| Generation of SAG1 driven *TgATG8* knockdown cell line | | |
| ML2669/  ML2670 | TTTATGCATGAAGATCCGATCTTGCTGCTG | TTTATGCATACAACCGTGTGTTTACACGAC |
| ML2477/  ML2664 | Gacaaggtcccgcgctgtcttcgctcgctagaagatccgatcttgctgct | CGAAGGACACTTCGTCGCGAATCGATGGCATTTTGTATAGTTCATCCATGCC |
| ML2885/  ML2886 | AAGTTGCGACGAATTCCTTTTCAGCG | AAAACGCTGAAAAGGAATTCGTCGCA |
| ML2667/ ML2666 | Gtagagcgcgtagttcgcc | CAGCGAGAGTCAATGGGACG |
| ML2667/ ML2668 | Gtagagcgcgtagttcgcc | GTGCTTACATCGAACACGG |
| ML2695/ ML1883 | TTCAAGACCCGCCACAACAT | ACACGTGCTGCTCATACACT |
| Targeted disruption and complementation of *TgATG9* | | |
| ML2465/  ML2466 | ATTAAGCTTgatcagcacgaaaccttgca | AAAGGATCCcctccaccgcggtgtca |
| ML2467/  ML2468 | AAGTTGTTGAATCTATAACCGTGCCG | AAAACGGCACGGTTATAGATTCAACA |
| ML1851/ ML2222 | ATTTTCAGTGACTCGAGGAGACGTC | GGGTTCTTGTGTCTTATGCC |
| ML2725/ ML2222 | GTGGACACAGTCGGTTGACA | GGGTTCTTGTGTCTTATGCC |
| ML2726/ ML2232 | GAATGCAAGGTTTCGTGCTGAT | GGAGACATTGACTATTACAG |
| DS5/  DS8 | CGAGGAGACGTCAACTGAAGATTGCGGCCGCACCTGTTTTTGG | CGTGATATGGTCCATATCATACGGGACCTGGGCTTTGCAGATAATTTTCC |
| DS6/  DS7 | GCAAAGCCCAGGTCCCGTATGATATGGACCATATCACGTCTTCATACC | CCGCAATCTTCAGTTGACGTCTCCTCGAGTCACTGAAAATGTCC |
| DS17/  DS18 | ATGCACATGTCGTCACATGCCAGTGGCAGTAACG | GAATCGACAGGCATCTGTCGCCTGAG |
| DS24/  DS25 | ACGTCCCGGACTACGCTGGCTATCCCTATGATGTGCCCGATTATGCGTATCCTTACGATGTTCCAGATTATGCCTGAAGATTGCGGCCGCACCTG | CACATCATAGGGATAGCCAGCGTAGTCCGGGACGTCGTACGGGTACCTAGGGAATTCCCGTCCTCCACTTCCGTTGACGTCTCCTCGAGTCACTG |
