# Supplementary material for: Toxoplasma TgATG9 is critical for autophagy and long-term persistence in tissue cysts
Source: eLife. 2021 Apr 27;10:e59384. doi: 10.7554/eLife.59384 (PMC8128441; doi:10.7554/eLife.59384)
Supplement: Supplementary file 2. [file elife-59384-supp2.docx]

Supplementary File 2. CellProfiler parameters for determining mitochondria morphology

| **Measurements used to define mitochondria morphology** |
| --- |
| Granularity_11_F1ATPase_Cyst |
| Granularity_16_F1ATPase_Cyst |
| Granularity_2_F1ATPase_Cyst |
| Granularity_8_F1ATPase_Cyst |
| Texture_AngularSecondMoment_F1ATPase_5_03_256_Cyst |
| Texture_Contrast_F1ATPase_20_02_256_Cyst |
| Texture_Contrast_F1ATPase_40_03_256_Cyst |
| Texture_Correlation_F1ATPase_20_00_256_Cyst |
| Texture_Correlation_F1ATPase_20_02_256_Cyst |
| Texture_Correlation_F1ATPase_40_02_256_Cyst |
| Texture_Correlation_F1ATPase_5_00_256_Cyst |
| Texture_Correlation_F1ATPase_5_01_256_Cyst |
| Texture_Correlation_F1ATPase_5_02_256_Cyst |
| Texture_Correlation_F1ATPase_5_03_256_Cyst |
| Texture_DifferenceEntropy_F1ATPase_20_00_256_Cyst |
| Texture_DifferenceEntropy_F1ATPase_40_02_256_Cyst |
| Texture_DifferenceVariance_F1ATPase_5_00_256_Cyst |
| Texture_DifferenceVariance_F1ATPase_5_03_256_Cyst |
| Texture_InfoMeas1_F1ATPase_40_00_256_Cyst |
| Texture_InfoMeas1_F1ATPase_40_02_256_Cyst |
| Texture_InfoMeas1_F1ATPase_5_00_256_Cyst |
| Texture_InfoMeas1_F1ATPase_5_01_256_Cyst |
| Texture_InfoMeas1_F1ATPase_5_02_256_Cyst |
| Texture_InfoMeas1_F1ATPase_5_03_256_Cyst |
| Texture_InfoMeas2_F1ATPase_20_02_256_Cyst |
| Texture_InfoMeas2_F1ATPase_20_03_256_Cyst |
| Texture_InfoMeas2_F1ATPase_40_00_256_Cyst |
| Texture_InfoMeas2_F1ATPase_40_02_256_Cyst |
| Texture_InfoMeas2_F1ATPase_40_03_256_Cyst |
| Texture_InfoMeas2_F1ATPase_5_00_256_Cyst |
| Texture_InfoMeas2_F1ATPase_5_01_256_Cyst |
| Texture_InfoMeas2_F1ATPase_5_02_256_Cyst |
| Texture_InfoMeas2_F1ATPase_5_03_256_Cyst |
| Texture_InverseDifferenceMoment_F1ATPase_40_00_256_Cyst |
| Texture_InverseDifferenceMoment_F1ATPase_5_02_256_Cyst |
