# Supplementary material for: Toxoplasma TgATG9 is critical for autophagy and long-term persistence in tissue cysts
Source: eLife. 2021 Apr 27;10:e59384. doi: 10.7554/eLife.59384 (PMC8128441; doi:10.7554/eLife.59384)
Supplement: Transparent reporting form [file elife-59384-transrepform.pdf]

eLIFE
1st Floor
24 Hills Road
Cambridge CB2 1JP, UK
P 01223 855340
W elifesciences.org
T @elife

eLife's transparent reporting form

We encourage authors to provide detailed information *within their submission* to facilitate the interpretation and replication of experiments. Authors can upload supporting documentation to indicate the use of appropriate reporting guidelines for health-related research (see EQUATOR Network), life science research (see the BioSharing Information Resource), or the ARRIVE guidelines for reporting work involving animal research. Where applicable, authors should refer to any relevant reporting standards documents in this form.

If you have any questions, please consult our Journal Policies and/or contact us: editorial@elifesciences.org.

Sample-size estimation

- You should state whether an appropriate sample size was computed when the study was being designed
- You should state the statistical method of sample size computation and any required assumptions
- If no explicit power analysis was used, you should describe how you decided what sample (replicate) size (number) to use

Please outline where this information can be found within the submission (e.g., sections or figure legends), or explain why this information doesn't apply to your submission:

As stated in the methods section (page 28); "Power analysis was not performed when the study was designed. Sample sizes were determined based on the capacity of each particular assay to collect a sufficient number of values to support rigorous analyses using standard statistical tests".

Replicates

- You should report how often each experiment was performed
- You should include a definition of biological versus technical replication
- The data obtained should be provided and sufficient information should be provided to indicate the number of independent biological and/or technical replicates
- If you encountered any outliers, you should describe how these were handled
- Criteria for exclusion/inclusion of data should be clearly stated
- High-throughput sequence data should be uploaded before submission, with a private link for reviewers provided (these are available from both GEO and ArrayExpress)

Please outline where this information can be found within the submission (e.g., sections or figure legends), or explain why this information doesn't apply to your submission:

eLife Sciences Publications, Ltd is a limited liability non-profit non-stock corporation incorporated in the State of Delaware, USA, with company number 5030732, and is registered in the UK with company number FC030576 and branch number BR015634 at the address 1st Floor, 24 Hills Road, Cambridge CB2 1JP | August 2014

The number of biological and technical replicates are stated in each figure legend, in conjunction with reference to the figure panel that represents the associated data.
Where quantifiable data was recorded, a minimum of 3 biological replicates were performed. The only exception to this being TEM data, which was performed once (data in table 1 gained from 144-235 technical replicates - individual bradyzoites from within 33-60 cysts).
In one particular case (dark puncta measurements), the number of biological replicates was increased to 6 due to the availability of data (images that could be analyzed) from two separate experiments.
Four biological replicates were used for the bradyzoite plaque assay in figure 7C.
For plaque assays, 3 technical replicates (plate wells for plaquing) per biological replicate (single experiment) were used.
It is stated in figure legends where data points represent individual technical replicates, as well as stating the number of biological replicates included in the study.
The number of mice per sample group for *in vivo* experiments is reported in the relevant methods section.
qRT-PCR analysis to determine *Atg9* expression levels was performed twice (cDNA generated from different parasite cultures in each case).
Data were analyzed after removing outliers using ROUT with a Q value of 0.1%.

eLIFE
1st Floor
24 Hills Road
Cambridge CB2 1JP, UK
P 01223 855340
W elifesciences.org
T @elife

Statistical reporting

- Statistical analysis methods should be described and justified
- Raw data should be presented in figures whenever informative to do so (typically when N per group is less than 10)
- For each experiment, you should identify the statistical tests used, exact values of N, definitions of center, methods of multiple test correction, and dispersion and precision measures (e.g., mean, median, SD, SEM, confidence intervals; and, for the major substantive results, a measure of effect size (e.g., Pearson's r, Cohen's d)
- Report exact p-values wherever possible alongside the summary statistics and 95% confidence intervals. These should be reported for all key questions and not only when the p-value is less than 0.05.

Please outline where this information can be found within the submission (e.g., sections or figure legends), or explain why this information doesn't apply to your submission:

A paragraph on statistical analysis is included at the end of the methods section.
Values of N are included in figure legends where relevant.
Information pertinent to what data points and bars on graphs represents (e.g. mean, SD, SEM) is also stated in the respective figure legends for that panel.
Significantly different samples are denoted on figures with asterisks and the p value associated with them is stated in the respective figure legends.

(For large datasets, or papers with a very large number of statistical tests, you may upload a single table file with tests, Ns, etc., with reference to sections in the manuscript.)

Group allocation

- Indicate how samples were allocated into experimental groups (in the case of clinical studies, please specify allocation to treatment method); if randomization was used, please also state if restricted randomization was applied
- Indicate if masking was used during group allocation, data collection and/or data analysis

Please outline where this information can be found within the submission (e.g., sections or figure legends), or explain why this information doesn't apply to your submission:

Mice were randomly allocated into groups prior to infection with *T. gondii*. Samples were then grouped based on the strain of *T. gondii* they were injected with IP.
Samples were blinded prior to ex vivo cyst counts.

Additional data files ("source data")

- We encourage you to upload relevant additional data files, such as numerical data that are represented as a graph in a figure, or as a summary table
- Where provided, these should be in the most useful format, and they can be uploaded as "Source data" files linked to a main figure or table
- Include model definition files including the full list of parameters used
- Include code used for data analysis (e.g., R, MatLab)
- Avoid stating that data files are "available upon request"

Please indicate the figures or tables for which source data files have been provided:

eLife Sciences Publications, Ltd is a limited liability non-profit non-stock corporation incorporated in the State of Delaware, USA, with company number 5030732, and is registered in the UK with company number FC030576 and branch number BR015634 at the address 1st Floor, 24 Hills Road, Cambridge CB2 1JP | August 2014

eLife Sciences Publications, Ltd is a limited liability non-profit non-stock corporation incorporated in the State of Delaware, USA, with company number 5030732, and is registered in the UK with company number FC030576 and branch number BR015634 at the address 1st Floor, 24 Hills Road, Cambridge CB2 1JP | August 2014
